# Ferroptosis Inhibition with Deferoxamine Alleviates Radiation-Induced Fibrosis

**DOI:** 10.21203/rs.3.rs-4314380/v1

**Published:** 2024-05-27

**Authors:** Charlotte E. Berry, Carter Kendig, Thalia Le BS, Camille Brenac, Michelle Griffin, Jason Guo, Lionel Kameni, Scott J. Dixon, Michael T. Longaker, Derrick Wan

**Affiliations:** Stanford University; Stanford University; Stanford University; Stanford University; Stanford University; Stanford University; Stanford University; Stanford University; Stanford University; Stanford University

**Keywords:** ferroptosis, deferoxamine, radiation-induced brosis, ionizing radiation, wound healing

## Abstract

**Background:**

Radiation-induced fibrosis (RIF) is a debilitating sequelae of radiation therapy that has been shown to improve with topical treatment with the iron chelator deferoxamine (DFO). We investigated whether DFO exerts this effect through attenuation of ferroptosis, a recently described iron-dependent pathway of cell death.

**Methods:**

Adult C57BL/6J mice were treated with topical DFO or ferrostastin-1 (Fer-1) and irradiated with 30 Grays of ionizing radiation to the dorsal skin to promote development of chronic RIF. Immunofluorescent staining with 4-hydroxynonenal (4-HNE) antibody was carried out directly following irradiation to assess ferroptosis activity. Perfusion testing with laser Doppler was performed throughout the healing interval. Eight weeks following radiation, dorsal skin was harvested and analyzed histologically and biomechanically.

**Results:**

Immunohistochemical staining demonstrated lower presence of 4-HNE in non-irradiated skin, DFO-treated skin, and Fer-1-treated skin compared to irradiated, untreated skin. DFO resulted in histological measurements (dermal thickness and collagen content) that resembled normal skin, while Fer-1 treatment yielded less significant improvements. These results were mirrored by analysis of extracellular matrix ultrastructure and biomechanical testing, which recapitulated the ability of topical DFO treatment to alleviate RIF across these parameters while Fer-1 resulted in less notable improvement. Finally, perfusion levels in DFO treated irradiated skin were similar to measurements in normal skin, while Fer-1 treatment did not impact this feature.

**Conclusions:**

Ferroptosis contributes to the development of RIF and attenuation of this process leads to reduced skin injury. DFO further improves RIF through additional enhancement of perfusion not seen with Fer-1.

## Background

More than half of patients treated for cancer receive radiation therapy as part of their treatment regimen. (1) While this treatment can be life-saving, tissues within the therapeutic field are exposed to ionizing radiation and are at risk for the development of associated complications.(1–3) With healthy skin is subjected to radiation, subsequent radiation-induced fibrosis (RIF) can occur which significantly impacts quality of life.

Typically emerging three or more months following radiation therapy, cutaneous RIF is characterized by signs and symptoms including skin retraction and induration, pain, necrosis, ulceration, and restricted range of motion. Ionizing radiation damage occurs through direct DNA damage and the generation of reactive oxygen species (ROS) from water. Initial radiation injury prompts an acute inflammatory response that incites the recruitment of fibroblasts and subsequent excessive deposition of extracellular matrix (ECM).(4) The cellular mechanisms that underlie this process continue to be characterized in recent years, with particular interest emerging in a novel type of cell death known as ferroptosis.

Ferroptosis was described in 2012 as an iron-dependent cell death mechanism that is driven by high levels of lipid peroxide accumulation.(5) Morphologically, ferroptosis is characterized by mitochondrial shrinkage, increased membrane density, and disappearance or reduction in visibility of the mitochondrial cristae.(5) These phenotypes vary significantly from the widely recognized characteristics of necrosis, such as cytoplasmic swelling and cell membrane rupture, and apoptosis, with cell shrinkage, chromatin condensation, and cytoskeletal disintegration.(6)

Ferroptosis can be triggered biochemically by the depletion of intracellular cysteine, and cysteine-containing metabolites including GSH, and a decrease in lipid hydroperoxide reduction to lipid alcohols by glutathione peroxidase 4. Iron can participate in the initiation and propagation of lipid peroxidation in the membrane, and when this process runs unopposed the end result is ferroptotic cell death.(7, 8) Ferroptosis is regulated by genes related to iron and lipid metabolism. Questions remain regarding the specific genetic regulatory mechanism of ferroptosis, and a thorough understanding of the cellular interactions underlying ferroptosis requires further research.

Since the recognition of this novel mode of cell death, significant interest has emerged in how ferroptosis contributes to critical regulatory pathways and pathologic conditions. Ferroptosis may contribute to cancer cell death as well as off-target irradiation damage in a variety of tissue types, across hematopoietic, gastrointestinal, pulmonary, and cardiovascular systems.(9, 10) In relationship to cutaneous RIF, a 2021 study by Vats et al. demonstrated that ferroptosis underlies the pathogenesis of ultraviolet radiation-driven cutaneous inflammation.(11)

Recent studies have shown that the iron chelator deferoxamine (DFO) demonstrates therapeutic potential in the attenuation of cutaneous RIF via topical treatment.(12–14) DFO’s efficacy has often been attributed to the agent’s ability to stabilize the pro-angiogenic transcription factor hypoxia inducible factor 1 subunit alpha (HIF1α) by limiting iron-dependent degradation.(15–17) Promotion of angiogenesis in the context of radiation-mediated endothelial damage improves oxygen and nutrient delivery to the wounded tissue. Secondarily, DFO may also treat cutaneous RIF by decreasing oxidative damage by ROS which accrue through Fenton-based chemistry dependent on ferric iron as a catalyst, thereby decreasing iron-dependent cell death.(18, 19) Given DFO’s documented efficacy in treating cutaneous RIF and the ability of the drug to chelate iron, we sought to investigate the contribution of each of these effects on DFO’s ability to improve RIF.

## Methods

### Animals

**Figure 1:**
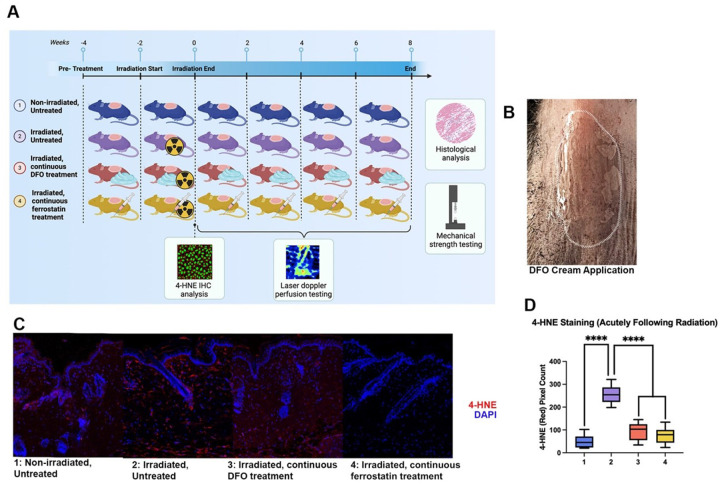
Schematic overview of the study protocol, timelines, and analyses and ferroptosis changes in irradiated skin. **Caption: A** Schematic of mouse allocation across experimental conditions and analyses performed during different timelines **B.** Application of deferoxamine cream on mouse dorsum **C.** Histological representation of 4-HNE staining for all treatment conditions **D.** Quantification of 4-HNE immunofluorescent staining revealed an increase acutely following irradiation (Group 2) that was decreased by both DFO treatment (Group 3) and Ferrostatin treatment (Group 4) to levels that were not significantly different from those seen in normal skin (Group 1).

Female C57BL/6 mice aged 8 weeks (The Jackson Laboratory, Bar Harbor, ME) were separated into four experimental groups (n = 8 mice/group): 1) Untreated non-irradiated, 2) Untreated irradiated, 3) Irradiated with DFO treatment, and 4) Irradiated with ferrostatin (Fer-1) treatment (Fig. 1A). Untreated, non-irradiated mice (Group 1) did not receive radiation. Untreated irradiated mice (Group 2) underwent radiation but did not receive any topical DFO or Fer-1 injection. Mice in the irradiated with DFO treatment group (Group 3) received two weeks of daily topical DFO treatment before undergoing the irradiation protocol. Daily DFO treatment for this group continued throughout the administration of the irradiation protocol. Irradiated mice with continuous Fer-1 treatment (Group 4) received two weeks of Fer-1 treatment before undergoing the irradiation protocol in addition to continued Fer-1 treatment for the remainder of the experiment.

Mice were housed in sterile micro-insulators at the Research Animal Facility, with five animals per cage. They had unrestricted access to water and rodent chow, adhering to appropriate guidelines. All experiments were conducted in compliance with an approved APLAC protocol (APLAC No. 31212) and followed the guidelines of the University Animal Care and Use Committee.

### Deferoxamine administration

DFO was purchased in a topical cream formulation (TauTona Group, Redwood City, CA) and applied to the dorsi of mice in Group 3. The cream was created at a concentration of 100mg DFO per 15g of cream. The cream was administered in 450 mg aliquots and spread in a 1.5×2 cm rectangular area to cover the entire irradiated field of dorsal skin (Fig. 1B). To control for the effects of the cream formulation itself, all other groups (1, 2, and 4) received the same treatment regimen with DFO-free topical ointment utilizing the same formulation and created by the same manufacturer.

### Ferrostatin administration

The ferroptosis inhibitor, Fer-1, was procured from Selleck Chemicals (S7243, TX, United States) and dosing followed established protocols.(20, 21) For animals in Group 4, Fer-1 was delivered intraperitoneally (i.p., 2.5 mg/kg/day in normal saline) throughout the experimental protocol, commencing two weeks before the start of irradiation. All other groups (1, 2, and 3) received equal volumes of normal saline solution injections, adhering to the same injection schedule.

### Irradiation protocol

Prior to irradiation, the dorsal skin was shaved with clippers and treated with Nair^™^ depilatory cream. A cumulative dose of 30 Grays (Gy) from external beam radiation, mirroring the standard whole breast radiation therapy for humans, was administered to the dorsum of the mice. This radiation was delivered in six fractionated doses of 5 Gy over a span of 12 days using the Kimtron Polaris SC-500 system from Kimtron, Inc. (Oxford, CT).(14) Lead shielding was employed to safeguard all regions of the mouse, excluding the dorsum. After the conclusion of the radiation protocol, four weeks of time elapsed to allow for RIF to develop.(12, 13)

### Mouse tissue harvest

Mouse dorsal skin was harvested following the completion of radiation (n = 3 mice/group) or at the conclusion of the experimental protocol eight weeks following the conclusion of the irradiation protocol (n = 5 mice/group). Samples designated for histological analysis were immersed in 10% neutral buffered formalin overnight, processed, paraffin-embedded, and cut into sections of 6-μM thickness. Samples intended for mechanical strength testing were fashioned into full-thickness strips, with the previously irradiated area positioned centrally and tapered. This ensured that no normal skin was present around the specified region. The strips were preserved in Dulbecco’s Modified Eagle Medium (ThermoFisher Scientific) on ice until testing.

### Histology

#### 4-HNE immunohistochemical analysis:

4-HNE immunofluorescent staining was performed on histologic sections of each skin sample. Incubation was performed with anti-4-HNE primary antibody (1:50; MA5-27570; Invitrogen) followed by an Alexa Fluor 647-conjugated donkey anti-rabbit IgG secondary antibody (1:500, ab150075; Abcam). Red pixel area was obtained from 20 X magnification images (n = 15 per condition) via ImageJ (NIH) analysis that recognized red hues, binarized the images, and counted selected pixels.(22)

#### Assessment of dermal thickness and collagen density

To assess dermal thickness, sections of murine skin underwent hematoxylin and eosin (H&E) staining (Cat. No. H-3502; Vector Laboratories, Burlingame, CA). Collagen density was evaluated by staining specimens with Masson’s Trichrome (MT) (ab150686; Abcam, Cambridge, United Kingdom). The dermis, characterized as the vertical distance from the basal layer of the epidermis to the underlying hypodermis, was measured in randomly selected sections for each condition using a Leica DMI4000 B microscope (Leica Microsystems, Wetzlar, Germany) at the 10X and 20X objective, ensuring robust statistical power for analyses.

Images of MT-stained skin were taken at the 10X and 20X objectives. Integrated density measurements of stained collagen were derived from the same 10 selected sections per condition using the ImageJ color deconvolution plugin. The quantity of blue pixels was quantified through ImageJ using a Color Detect macro.

#### Collagen fiber network analysis

To analyze fiber networks, sections were subjected to Picrosirius Red staining (ab150681; Abcam) using standard protocols. Picrosirius-stained skin samples were captured at 40X magnification under a polarized light source with a Leica DM5000 B light microscope (Leica Microsystems) at the 40X objective (100 images per condition). The images of Picrosirius Red-stained slides underwent color deconvolution, were converted to grayscale, binarized, and skeletonized using an algorithm in MATLAB.

Characteristics of collagen fibers, encompassing aspects of maturation and organization (such as length, width, branch points, brightness, number, persistence, angle, Euler number, extent, perimeter, solidity, eccentricity, equivalent diameter), were derived from the skeletonized images. To simplify the data, dimensionality reduction techniques were applied, resulting in the creation of two-dimensional t-distributed stochastic neighbor embedding plots. These plots served as visual representations, effectively illustrating distinctions in collagen fiber network patterns among various groups, as detailed in a previous study.(23)

#### Skin biomechanical testing

Tissue samples from mice, collected during harvesting, were loaded into an MTS Bionix 200 (MTS Systems, Eden Prairie, MN) fitted with an Interface SM-10 force transducer. The dimensions of each scar, including length, width, and thickness, were measured using calipers. Mechanical strength testing was conducted at a rate of 100 μm/s. Stress–strain curves were generated, and tensile strength was calculated using Matlab (Mathworks, Natick, MA) based on the collected data, adjusting for the length, width, and thickness of each tissue sample.

#### Laser Doppler for skin vascularity

Skin perfusion was monitored biweekly on the dorsum following completion of radiation until the conclusion of the experimental duration. Laser Doppler perfusion was assessed with a PeriScan PIM 3 (Perimed, Las Vegas, NV). The mean perfusion within the 1.5 × 2 cm treatment field was recorded twice for each mouse through consecutive scans. The scans were conducted under inhaled anesthesia, with a heating pad placed beneath the induction chamber to maintain a consistent ambient room temperature of 73°F.

#### Statistical analysis

The data are presented as means and error bars indicate the standard deviation. Parametric analyses involved two-tailed Student’s t-tests for two-group comparisons and one-way analysis of variance, followed by Tukey’s multiple-comparisons test for multiple groups. Nonparametric analyses utilized the Kruskal–Wallis test with post-hoc Dunn’s testing to compare means among groups. All statistical analyses were conducted using GraphPad Prism (GraphPad Software, San Diego, CA). A significance level of *p < 0.05 was considered statistically significant.

## Results

### Immunohistochemical ferroptosis marker evaluation

To determine how DFO may affect ferroptosis in irradiated skin, mice were treated before, during, and after radiation therapy. Mice were similarly treated with Fer-1, a small molecule lipophilic radical trapping antioxidant known to potently and selectively inhibit ferroptosis. Immunofluorescent staining revealed that both DFO and Fer-1 treatment effectively decreased quantities of 4-HNE, a common marker of ferroptosis, in the murine dermis acutely following irradiation. There was no significant difference in 4-HNE levels between murine skin treated with DFO or Fer-1 and normal skin. Conversely, in irradiated, untreated mice, 4-HNE levels were significantly greater at this time point (Fig. 1C–D).

### Dermal thickness and collagen deposition

**Figure 2:**
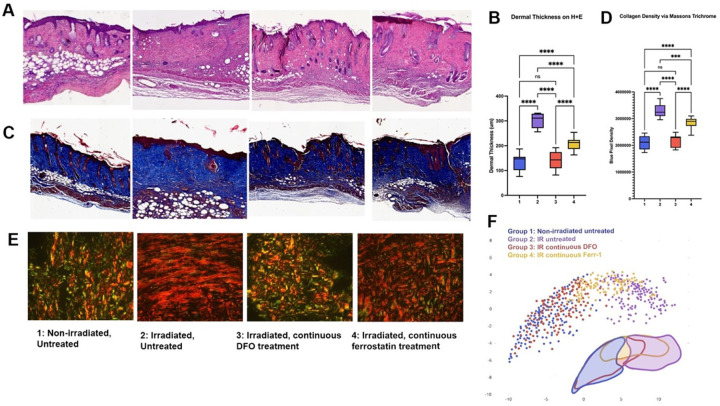
Histological analysis of skin with quantitative analysis. Caption: A. Histological representation of Hematoxylin and Eosin (H&E) staining for all treatment conditions. B. Quantification of dermal thickness for each group via H&E staining demonstrated no significant difference between normal skin (Group 1) and DFO-treated skin (Group 3). Ferrostatin treatment (Group 4) resulted in a thinner dermis than irradiated, untreated skin (Group 2) (****p<0.0001), but a thicker dermis than normal skin (Group 1) (****p<0.0001). C. Histological representation of Masson’s Trichrome (MT) staining for all treatment conditions. D. Quantification of blue pixel density for each group via MT staining demonstrated no significant difference between normal skin (Group 1) and DFO-treated skin (Group 3). Ferrostatin treatment (Group 4) resulted in a thinner dermis than irradiated, untreated skin (Group 2) (**p<0.01), but a thicker dermis than normal skin (Group 1) (****p<0.0001). E. Histological representation of Picrosirius Red staining for all treatment conditions. F. Machine-learning algorithm-derived collagen ultrastructure UMAP representation of dermal extracellular matrix showed a significant overlap of normal skin (Group 1) and DFO treated groups (Group 3). Ferrostatin-treated skin (Group 4) appeared to more closely resemble Irradiated Untreated skin (Group 2).

To further explore the effect of DFO therapy and Fer-1 treatment in irradiated skin, we assessed the dermal thickness and collagen density of skin in each treatment condition eight weeks following completion of radiation. Dermal thickness was assessed via H&E staining and revealed that DFO treatment (Group 3) restored dermal thickness in irradiated skin to a similar level observed in the non-irradiated skin. Fer-1 treatment (Group 4) also improved dermal thickness, with significantly thinner thickness than untreated, irradiated skin (Group 2), though improvement was less than what was appreciated with DFO (Fig. 2A–B). The collagen density of each treatment condition assessed by MT staining showed that DFO treatment had similar collagen content to untreated, non-irradiated skin, mirroring the H&E findings. As with dermal thickness, Fer-1 also resulted in improvement in collagen density over untreated, irradiated skin, however this was less significant than DFO (Fig. 2C–D).

### Collagen structure

Picrosirius Red staining was used to assess the collagen fiber assembly and extracellular matrix ultrastructure of the irradiated skin. Analysis of these features using a supervised machine learning algorithm demonstrated that DFO-treated skin had features which clustered more similar to normal skin and were distinct from Fer-1-treated skin and irradiated, untreated skin (Fig. 2E–F). Fer-1-treated skin appeared to cluster intermediately between normal skin and irradiated, untreated skin (Fig. 2E–F).

### Skin perfusion

**Figure 3:**
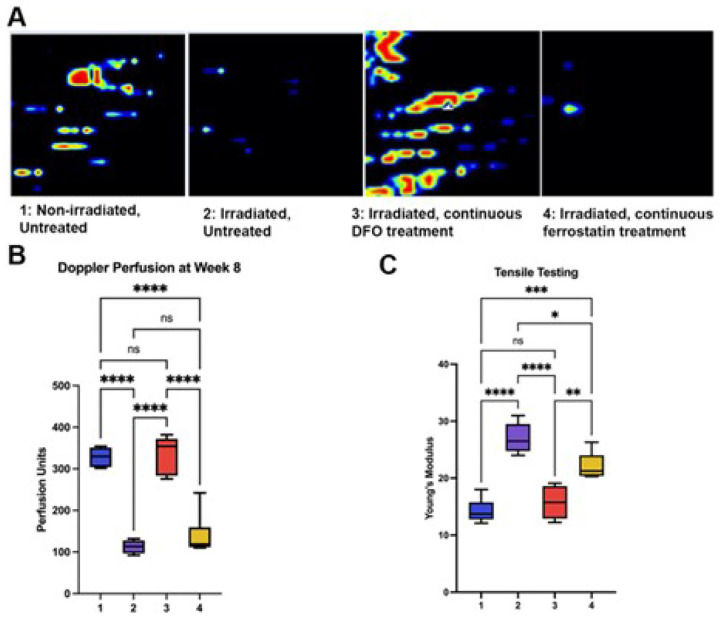
Perfusion analysis and biomechanical testing. **Caption: A.** Heat map representative scans of the mouse dorsum at week 8 for each treatment group. Black/dark blue colors represent lower perfusion, and the yellow/red colors represent higher perfusion **B.** Quantification of laser Doppler perfusion index demonstrated that at week 8, there was no significant difference between normal skin (Group 1) and DFO-treated skin (Groups 3). There was also no significant difference found between irradiated, untreated skin (Group 2) and Ferrostatin-treated skin (Group 4) at this time point **C.** Quantification of Young’s modulus via tensile testing demonstrated that at week 8, there was no significant difference between normal skin (Group 1) and DFO-treated skin (Group 3). There was also no significant difference found between irradiated, untreated skin (Group 2) and Ferrostatin-treated skin (Group 4) at this time point.

As cutaneous RIF is known to result in decreased perfusion, laser Doppler analysis was employed serially following completion of radiation treatment to measure this clinically relevant parameter. In concordance with previous results, longitudinal laser Doppler perfusion measurements revealed an initial short-term rise in perfusion two weeks following IR, followed by steady and significant decrease in the perfusion of irradiated skin through the conclusion of the experimental timeline. However, at 8 weeks following completion of radiation, there was no significant difference between normal skin and DFO-treated skin. In contrast, Fer-1 treatment did not impact perfusion, as laser Doppler measurements in this group were similar to untreated, irradiated skin (Fig. 3A–B).

### Skin biomechanics

Paralleling perfusion findings, tensile testing of skin samples at week 8 demonstrated that DFO treatment resulted in a biomechanical profile not significantly different from normal skin. At the 8-week timepoint, there was no significant difference between normal skin and DFO-treated skin stiffness (Fig. 3C). Skin stiffness also improved with Fer-1 treatment, though measurements showed this was less significant than seen with DFO. (Fig. 3C).

## Discussion

Radiation therapy represents a common therapeutic approach for many forms of cancer, of which there are nearly 2 million new cases diagnosed each year.(24) As improvements in cancer therapy have increased the average length of cancer survival, a growing number of survivors are living with long-term sequelae of radiation therapy such as RIF. Shown to have a profound impact on long-term quality of life, RIF can lead to severe cosmetic and functional impairment.(25, 26) At the tissue and cellular levels, RIF manifests as epidermal thinning, eosinophilic homogenized sclerosis of dermal collagen, presence of scattered large and atypical fibroblasts, and fibrous thickening leading to luminal obliteration of deep vessels.(25–27) Dermal thickening paired with vascular damage results in an environment in which wound healing is impaired, leading to a uniquely challenging setting for surgical reconstruction.(25)

Despite the clinical significance and the rising incidence of RIF, the current array of therapeutic options remains restricted, particularly in the realm of topical treatments. Presently, patients have access to treatments such as physical therapy, fat grafting, and vitamin E. However, the limited and mixed evidence, coupled with logistical barriers and high costs, has hindered the widespread adoption of these options. Topical DFO has emerged as a treatment for RIF, and has previously been shown to attenuate cutaneous RIF in a murine model across biomechanical and histological measurements as well as improve perfusion to the skin.(12, 13)

To investigate the cellular mechanisms underlying the demonstrated efficacy of DFO, this study focused on the effect DFO treatment may have on ferroptosis, an iron-dependent mechanism of cellular death, which has recently been tied to IR-induced damage in a variety of tissue types.(28–31) Historically, DFO has also been known to restore vascularity by stabilizing HIF1α through chelation of iron, an integral co-factor necessary for prolyl hydroxylase domain-containing protein 2-mediated degradation of HIF1α. Stabilization of HIF1α leads to an increase in downstream angiogenic factors and recruitment of endothelial progenitor cells.(32, 33) In addition to this pathway, a previous study has suggested that DFO may act through additional cellular pathways to impart a therapeutic effect.(14)

This study is the first, to our knowledge, to demonstrate *in vivo* the occurrence of ferroptosis in skin following ionizing radiation injury. We observed that topical DFO treatment reduces a well-established marker of ferroptosis, 4-HNE, to levels comparable to that achieved by Fer-1, consistent with cutaneous inhibition of ferroptosis. In the case of Fer-1, this finding mirrors previous studies where IP injection has been proven effective at inhibiting ferroptosis in a variety of organ systems.(20, 34) DFO has also demonstrated this capability *in vitro*,(11) via IP injection,(35) and intraarticular injection,(36) though never before as a topical treatment.

Our findings show that DFO treatment resulted in tissue-level alteration measured by histology that indicate the prevention and/or alleviation of dermal architectural changes known to characterize RIF. Including reduced dermal thickness measured by H&E and reduced collagen density measured by MT staining, these results recapitulate some previous findings which have demonstrated the ability of topical DFO treatment to attenuate RIF across these parameters.(12) Notably, Fer-1 treatment was found to moderately improve these histological measures of RIF as well, though not as much as DFO treatment. This finding was mirrored in other outcome measures as well, including extracellular matrix ultrastructure analysis and biomechanical testing, with measured parameters more similar to that of normal skin, but not to the degree achieved by DFO.

Perfusion imaging with laser Doppler confirmed that topical DFO treatment minimizes hypoperfusion of the dermis characteristic of chronic RIF,(12–14) while Fer-1 treatment did not. Taken together, these results indicate that while topical DFO treatment may inhibit ferroptosis, this effect alone does not explain the full therapeutic action of the iron chelator. As discussed above, the ability of DFO to promote perfusion through HIF1α stabilization has been previously described and may account for other differences, as Fer-1 is not known to interact with the VEGF pathway and has not been demonstrated to support neovascularization.

Notably, the safety profile of Fer-1 is a topic of current investigation, as several studies have indicated that the drug may have therapeutic potential in a variety of clinical settings, such as acute kidney and lung injury as well as cardiovascular disease.(37–39) However, some studies have indicated concern for liver toxicity, induction of autophagy, and immunosuppression.(7, 40, 41)

While our findings in a murine model show promise, additional experimentation is required to assess the potential translation of topical DFO treatment into clinical practice. Delving deeper into the intracellular effects of DFO may offer additional insights to explain the observed differences in outcome measures between Fer-1 and DFO treatments. While Fer-1 acts as an antioxidant and inhibits ferrous iron and lipid hypdroperoxide-dependent peroxidation, DFO chelates iron directly. Paired with the delivery of DFO through a reverse micelle formulation which allows for penetrance of the stratum corneum and perhaps intracellular entry,(12) this difference in mechanism may also account for some of our results indicating that DFO more effectively rescues RIF of the skin and restores perfusion compared to Fer-1 alone.

As DFO is known to promote angiogenesis through the stabilization of HIF1α, a theoretical concern exists regarding the use of this agent in sites where oncologic pathology may present. However, no studies to our knowledge have demonstrated an increased risk for cancer growth, metastasis, or recurrence following local administration of DFO. Furthermore, iron is known to participate in critical cellular functions such as oxygen transport, metabolism, and cell growth, and evidence has suggested that DFO may thus impart an anti-tumor effect.(14, 42, 43) Some tumors, in fact, have demonstrated iron dependency making them vulnerable to iron chelation by agents such as DFO.(44)

Notably, the formation of fibrosis in murine skin differs from that of humans in clinically relevant ways. For example, murine skin is known to heal and brose more rapidly than human skin, and time points analyzed for chronic fibrosis in mice were based on previously published studies.(12, 45, 46) Mouse skin varies from humans morphologically, as well, containing layers of differing relative thickness and the addition of a layer of subdermal muscle called the panniculus carnosis.(47) For these reasons, further investigation of this topic in large animal models such as pigs, which offer a skin structure more similar to that of humans, would be of substantial translational value.

## Conclusions

Cutaneous RIF is a growing clinical pathology that has a substantial impact on patient quality of life. While several clinical treatments, including massage and laser therapy, hyperbaric oxygen, pentoxifylline, and vitamin E have been studied and employed in an effort to attenuate the negative effects of RIF, these options have demonstrated mixed experimental efficacy.(48) Addressing this gap in care, topical DFO treatment offers ease of application paired with strong preclinical evidence. Collectively, our findings demonstrate the occurrence of ferroptosis in cutaneous RIF pathology, recapitulate the therapeutic potential of DFO, and suggest that DFO may alleviate RIF in part by its known capacity to promote angiogenesis, but also by reducing ferroptosis of skin cells in the irradiated field.

## Data Availability

The datasets used and/or analysed during the current study are available from the corresponding author on reasonable request.

## Abbreviations

DFO: Deferoxamine
ECM: Extracellular matrix
Fer-1: Ferrostatin
Gy: Grays
HIF1α: Hypoxia inducible factor 1 subunit alpha
H&E: Hematoxylin and Eosin
MT: Masson’s trichrome
RIF: Radiation-induced fibrosis
ROS: Reactive oxygen species
4-HNE: 4 Hydroxynonenal
